# Disease Detection or Public Opinion Reflection? Content Analysis of Tweets, Other Social Media, and Online Newspapers During the Measles Outbreak in the Netherlands in 2013

**DOI:** 10.2196/jmir.3863

**Published:** 2015-05-26

**Authors:** Liesbeth Mollema, Irene Anhai Harmsen, Emma Broekhuizen, Rutger Clijnk, Hester De Melker, Theo Paulussen, Gerjo Kok, Robert Ruiter, Enny Das

**Affiliations:** ^1^National Institute for Public Health and the EnvironmentCenter for Infectious Disease ControlBilthovenNetherlands; ^2^Maastricht UniversityDepartment of Work and Social PsychologyMaastrichtNetherlands; ^3^Radboud University NijmegenCentre for Language StudiesNijmegenNetherlands; ^4^Netherlands Organization for Applied Scientific Research (TNO)Healthy LivingLeidenNetherlands

**Keywords:** Internet, Web 2.0, measles, infectious disease outbreak, Netherlands, vaccination

## Abstract

**Background:**

In May 2013, a measles outbreak began in the Netherlands among Orthodox Protestants who often refuse vaccination for religious reasons.

**Objective:**

Our aim was to compare the number of messages expressed on Twitter and other social media during the measles outbreak with the number of online news articles and the number of reported measles cases to answer the question if and when social media reflect public opinion patterns versus disease patterns.

**Methods:**

We analyzed measles-related tweets, other social media messages, and online newspaper articles over a 7-month period (April 15 to November 11, 2013) with regard to topic and sentiment. Thematic analysis was used to structure and analyze the topics.

**Results:**

There was a stronger correlation between the weekly number of social media messages and the weekly number of online news articles (*P*<.001 for both tweets and other social media messages) than between the weekly number of social media messages and the weekly number of reported measles cases (*P*=.003 and *P*=.048 for tweets and other social media messages, respectively), especially after the summer break. All data sources showed 3 large peaks, possibly triggered by announcements about the measles outbreak by the Dutch National Institute for Public Health and the Environment and statements made by well-known politicians. Most messages informed the public about the measles outbreak (ie, about the number of measles cases) (93/165, 56.4%) followed by messages about preventive measures taken to control the measles spread (47/132, 35.6%). The leading opinion expressed was frustration regarding people who do not vaccinate because of religious reasons (42/88, 48%).

**Conclusions:**

The monitoring of online (social) media might be useful for improving communication policies aiming to preserve vaccination acceptability among the general public. Data extracted from online (social) media provide insight into the opinions that are at a certain moment salient among the public, which enables public health institutes to respond immediately and appropriately to those public concerns. More research is required to develop an automatic coding system that captures content and user’s characteristics that are most relevant to the diseases within the National Immunization Program and related public health events and can inform official responses.

## Introduction

In May 2013, a measles outbreak began in the Netherlands among Orthodox Protestants who often refuse vaccination for religious reasons [1].

In the Netherlands, the National Immunization Program (NIP) offers childhood vaccinations free of charge and vaccinations are not compulsory. Overall, the vaccination coverage among children up to age 4 years is high in the Netherlands and somewhat lower for boosters in children aged 4 and 9 years [2]. Since 1987, children have been offered vaccination against measles, mumps, and rubella in a 2-dose schedule at 14 months and 9 years of age, with a coverage of 96% and 93%, respectively [2]. Vaccination coverage among Orthodox Protestants was assessed in 2006-2008 and was found to be approximately 60% [3]. Orthodox Protestants are a close-knit community in the Netherlands, consisting of approximately 250,000 individuals. Predestination is an important doctrine in their belief and refusal of vaccination is based on the idea that people should not interfere with divine providence [4]. Other groups in the Netherlands that partly refuse measles vaccination include Anthroposophists and those with critical stances toward vaccination in general [5-6].

At the end of the outbreak, in February 2014, the incidence of measles was 0.16 per 1000 (2640 measles cases) in the Netherlands, resulting in more than 180 hospitalizations (approximately 7% of measles cases). In October 2013, a death occurred in a girl aged 17 years who was not vaccinated for religious reasons. The number of reported cases began to decline in October 2013 and at the end of February 2014, the National Institute for Public Health and the Environment (RIVM) announced the outbreak was over. Additional control measures implemented in July 2013, such as offering early vaccinations to children aged between 6 and 14 months of age living in municipalities with a low vaccination coverage (<90%), were ended.

The outbreak led to heated debates in traditional and social media. At the start of the measles outbreak, RIVM was asked for weekly updates of reported measles cases. In addition, some well-known politicians made public statements, such as “parents should take their responsibility and vaccinate their children” [7] and “clergymen should call for vaccinating their religious community” [8].

Outbreak patterns and related public opinions expressed in mass media channels are likely to diverge simply because not all epidemiological data are equally relevant in terms of news value. The traditional media agenda is determined by news value [9]; for example, the first severe case in an outbreak generally generates more media attention than later reported cases even if later cases are higher in numbers. Likewise, the start of an epidemic is generally more newsworthy than the peak of an epidemic because of the uncertainty involved at the beginning of an outbreak. Agenda-setting theory proposes that public opinion generally follows the (traditional) media agenda; the media does not determine what people think, but they do determine what people think about [10].

Social media can also be a rich source for researchers. Previous research has suggested a relationship between the number of influenza-related tweets and reported number of influenza-like illness [11-13]. Previous research has also suggested a relationship between social media and public opinion for influenza outbreaks such as H1N1 [13,14]. Results from these studies showed that H1N1-related tweets were primarily used to spread information from credible sources, but it also offered a platform for the exchange of opinions and experiences among the public. Variations in responses to different disease outbreaks was shown by Fung et al [15] who found the social media response among Chinese people to the H7N9 outbreak was 2-fold higher compared to the Middle East Respiratory Syndrome Coronavirus (MERS-CoV) outbreak. Therefore, analyzing social media appears useful in gaining insight into public opinion and/or disease patterns although it remains unclear to what extent previous findings generalize across different outbreaks.

Given that epidemiological patterns are likely to diverge from traditional media patterns and that previous social media research has focused on either disease detection or public opinion, the question remains if and when social media data reflect public opinion patterns versus disease patterns. This research aims to answer this question for the measles outbreak by investigating traditional and social media patterns across time and comparing these to reported measles cases. Our hypothesis is that because people generally use Twitter for spreading news and because the measles outbreak was highly publicized, the number of social media messages will show stronger convergence with the number of traditional media messages than with the epidemic curve (number of reported cases).

A second goal of this research was to analyze the content and specific sentiments expressed on Twitter, other social media, and online newspapers to detect factors that might affect intentions to vaccinate [16] and emotional states that might mediate vaccination behavior [17,18], and meaningful fluctuations in these factors over time. Jones et al [17] found that levels of anxiety waned along with the perception of the influenza A (H1N1) virus as an immediate threat. Translated to the measles outbreak, this research aimed to assess whether real-world events trigger significant increases or decreases in vaccination behavior-related sentiment such as expressed concern.

To summarize, adding to previous analyses of social media with regard to infectious disease outbreaks, this study aimed to compare the number of social media messages with the number of online news articles and with the epidemiological curve (ie, the number of reported measles cases) and assess the usefulness of social media in tracking factors that might affect vaccination behavior.

## Methods

### Data From Online (Social) Media

Because we were interested in discussions on online (social) media during the measles outbreak in the Netherlands in 2013, we used the search term “mazelen” (ie, measles; there are no synonyms used in the Dutch language) to select messages from online media. Data were gathered from April 15, 2013 (ie, 15 days before the start of the measles outbreak) to November 11, 2013 (ie, 14 days after the report of the measles-related death on October 28, 2013).

Articles from online newspapers were retrieved via LexisNexis and HowardsHome [19], tweets were retrieved via Twiqs.nl (free analytic Dutch tool for tweets [20]) and HowardsHome, and messages from other social media were also retrieved from HowardsHome. We could not get information about the data mining approaches used by the 2 companies LexisNexis and HowardsHome. For Twiqs.nl we used the data mining approach as has been described by Tjong Kim Sang [20]. Social media included messages from websites such as forums, weblogs, Facebook, and others (eg, advertisements, comments, information sites, status updates, podcasts, reviews/evaluation of products, social photo websites, social video websites, and wikis). For the selected articles in the newspapers (which were published online but were also available in paper version), all the national newspapers in the Netherlands were checked (ie, *NRC Handelsblad*, *De Volkskrant*, *Trouw*, *De Telegraaf*, *Algemeen Dagblad*, *Spits*, and *Metro*). We also included the religious-oriented newspapers *Reformatorisch Dagblad* and *Nederlands Dagblad*.

### Data on Measles Cases

Data on the number of measles cases were retrieved from the notification data of measles by the RIVM. The measles case definition of the European Centre for Disease Prevention and Control was used [21]. A measles case was defined if the person met the clinical criteria: fever and maculopapular rash and at least one of (1) cough, (2) coryza, or (3) conjunctivitis, and at least one of the laboratory criteria (1) isolation of measles virus from a clinical specimen, (2) detection of measles virus nucleic acid in a clinical specimen, (3) measles virus-specific antibody response in serum or saliva, or (4) detection of measles virus antigen by direct fluorescent antibody in a clinical specimen using measles-specific monoclonal antibodies (laboratory results need to be interpreted according to the vaccination status). A measles case could also be defined if the reported case did not meet the clinical and laboratory criteria but met the epidemiological criteria: an epidemiological link by human-to-human transmission (ie, contact less than 3 weeks ago with an identified measles case).

### Manual Topic and Sentiment Analyses

Data analysis was started by estimating the relative proportion of weekly online media messages and reported measles cases from April 15 to November 11, 2013, by scaling the numbers to the highest peak for all 4 data sources. The highest peak was assigned a score of 100. The reported measles cases by week of onset of exanthema were gathered to plot against the number of weekly media messages to see whether media followed the epidemiological curve. Tweets and retweets were analyzed together. To compare weekly number of online (social) media messages with one another and with weekly number of reported measles cases, Pearson correlations were calculated between the different sources using SAS 9.1.3 (SAS Institute Inc, Cary, NC, USA).

Furthermore, we analyzed the content of the messages (ie, topic) and how the messages were expressed (ie, sentiment). For each data source, the title was used for determining the topic and sentiment; if this was not clear or did not match with the summary, then the summary was used for determining the topic and sentiment. Note, for tweets, both title and summary contained the whole tweet. For newspaper articles and other social media messages retrieved via HowardsHome, the summary contained a maximum of 500 words. There was no minimum number of words. To identify the topics, thematic analysis was performed [22]. The process of coding and the development of themes were inductive in nature. A codebook was developed and initial codes provided various topics (n=25). On review and discussion, infrequently used (sub) topics were collapsed into larger (main) topics (n=8). Table 1 shows the topics and subtopics that emerged from the data with examples from tweets, other social media, and online newspapers.

The sentiments in the online newspaper articles generally differed from the sentiments in tweets and other social media messages. The sentiments for online newspaper articles fit better with objective nonjudgmental messages, whereas the sentiments for social media fit better with more personal and opinionated messages. Sentiments for online newspaper articles were, therefore, based on the classification used by Vasterman & Ruigrok [23], which included the following 3 sentiments: alarming (eg, “Teenager dead by measles infection”), reassuring (eg, “Start of extra vaccinations against measles”), and neutral / no sentiment / both alarming and reassuring (eg, “Measles epidemic has stabilized”). The sentiments for tweets and other social media messages were based on the article by Chew & Eysenbach [14], which emerged from analyzing their H1N1-related tweets. The sentiments included, among others, frustration, humor/sarcasm, concern, relief, question, minimized risk, information, and personal experiences. If the message contained more than one sentiment, the first sentiment identified was chosen. Table 2 shows examples of tweets and other social media messages for these various sentiments.

**Table 1:** Topics and subtopics (between parentheses) of tweets, other social media, and online newspapers about the measles outbreak or perceived risks.

Topic	Definition	Example (tweet, other social media, online newspaper)
Measles outbreak (including number of reported measles cases, measles deaths, people experiencing measles, and consequences of measles infection [including hospitalizations])	Objective information about the measles outbreak	“Number of measles cases has increased to 161” (online newspaper)
Refusing vaccination because of religious reasons	Opinions about persons refusing vaccination for religious reasons	“Unbelievable that the love for God can be greater than the love for your own child” (tweet)
Critical toward vaccination	Opinions about persons who are critical toward vaccination (eg, Anthroposophists)	“To remember: also followers of Rudolf Steiner (anthroposophical theory) and the Dutch society for conscientious vaccination are very much against vaccination! Also their children are taking a risk at getting measles” (other social media)
Perceived risks (including perceived severity of measles disease and not vaccinating against measles, adverse events, effectiveness of measles vaccine)	How public perceives risks of measles disease and measles vaccine	“That [ie, measles] was not that severe at all, I have experienced flu disease, which was much more severe” (other social media)
Measles prevention (including additional vaccinations, maternal measles antibodies, obligatory vaccination, vaccinating secretly, vaccinating employees, vaccinating religious people)	Preventive measures taken to control the measles spread	“Young adult without a measles vaccination cannot camp during summer” (tweet)
Trust and role institutions (including role of government, role of media, conspiracies)	No trust in information supply, should government interfere in whether people should vaccinate or not, and allegations about production of vaccines and vaccine components	“Subtle lies about measles by the RIVM? Naïveté?” (tweet)
Other	If it did not belong to one of the topics above	“What makes that the school exam and a measles infection are similar? Only children are affected!” (tweet)
Information not related to measles outbreak	If it had nothing to do with the measles outbreak or a relation with the measles outbreak could not be found	“The mortality of dolphins on the East coast of the USA is caused by a measles-related virus” (tweet)

**Table 2:** Sentiments of tweets and other social media messages about information or frustration.

Sentiment	Definition	Example (tweet/other social media)
Frustration	Tweet/message contains anger, irritation, contempt, criticism, or source is flabbergasted	“How stupid can you be by not vaccinating your children against measles” (tweet)
Humor/sarcasm	Tweet/message is funny or expresses sarcasm	“HAHAHAHAHAHA. He had drawn red spots on his head and said: ‘oooooh I have measles’” (tweet)
Concern	Tweet/message contains fear, concern, anxiety, worry, or grief about themselves or others	“Around me many vaccinated children with measles. A bit strange and alarming I think. Is there something known about this by the RIVM?” (tweet)
Relief	Tweet/message contains joy, happiness, or relief	“Thank God we are a liberal country (ie, that we have a choice to vaccinate or not)” (other social media)
Question	Tweet/message contains a question or questions for which the user would like to receive an answer	“This you probably know: what happens when you get measles? Do you need treatment or does it go away spontaneously” (tweet)
Minimized risk	Tweet/message minimizes the risk of measles infection and/or the possible complications	“That [ie, measles] was not that severe at all, I have experienced flu disease, which was much more severe” (other social media)
Information	Tweet/message contains information, informative retweets, and/or other information sources about measles	“RIVM expects more measles cases because school holidays are over” (tweet)
Personal experience	Tweet/message contains a personal experience/story about the disease without expressing any concerns	“My daughter has had encephalitis as a complication of an unknown virus infection” (other social media)
Other	Tweet contains none of the above 8 sentiments	“At the left wing also a number of persons are not vaccinating because of other reasons” (other social media)
Information not related to measles outbreak	Tweet has nothing to do with the measles outbreak or a relation with the measles outbreak could not be found	“The mortality of dolphins on the East coast of the USA is caused by a measles-related virus” (tweet)

For coding purposes, we limited the number of tweets and other social media messages by selecting every tenth tweet or message. This resulted in 2020 of 20,201 tweets in total and 552 of 5521 other social media messages in total. The number of tweets not related to the measles outbreak was 38 of 2020 (1.88%); therefore, the total number of tweets used for the analyses was 1982 of which 626 (31.58%) were retweets. The number of other social media messages unrelated to the measles outbreak was 88 of 5521 (15.94%); therefore, the total number of messages used for the analyses was 464. To be able to compare the topics of tweets with the topics of other social media messages and online newspaper articles, we again selected every tenth tweet of the 2020 tweets mentioned previously, which resulted in 202 tweets of which 6 tweets were not related to the measles outbreak and were excluded from the analysis. We analyzed retweets separately from tweets because retweets might provide insight into what people find interesting and important.

The topics and sentiments were coded for all measles-related online newspaper articles found (n=351). The number of online newspaper articles analyzed was 282 because 69 (19.7%) were unrelated to the measles outbreak. Of the 282 articles, 79 were published in the 2 religious-oriented newspapers and 203 were published in the 7 nonreligious-oriented newspapers. Both the topic and sentiment were only available for the articles in these 2 religious-oriented newspapers.

Each message was coded independently by 2 raters to establish coding reliability (ie, Cohen’s kappa with values <0 indicating no agreement, 0-.20 indicating slight agreement, .21-.40 indicating fair agreement, .41-.60 indicating moderate agreement, .61-.80 indicating substantial agreement, and .81-1 indicating almost perfect agreement [24]). During coding of the sentiments of tweets and coding of the sentiments and topics of other social media messages, there were some differences in insights between the raters. Regarding interpretation of tweets, one of the raters coded part of the tweets as concerned tweets whereas the other 2 raters (ie, each rater coded two-thirds of the tweets) coded these tweets as informative tweets. Regarding interpretation of other social media messages, one of the raters interpreted the other social media messages about refusing vaccination because of religious reasons differently compared to other raters. Another rater interpreted informative messages as neutral messages. After discussing these differences, informative messages (both tweets and other social media messages) and the other social media messages with the topic refusing vaccination because of religious reasons were recoded and the kappa was estimated. For tweets, the kappas for sentiment and topic were .79 and .77, respectively. For other social media, the kappas for sentiment and topic were .58 and .65, respectively. For online newspapers, the kappas for sentiment and topic were .80 and .81, respectively.

## Results

### Comparing Number of Measles Cases and Online (Social) Media Messages

**Figure 1:**
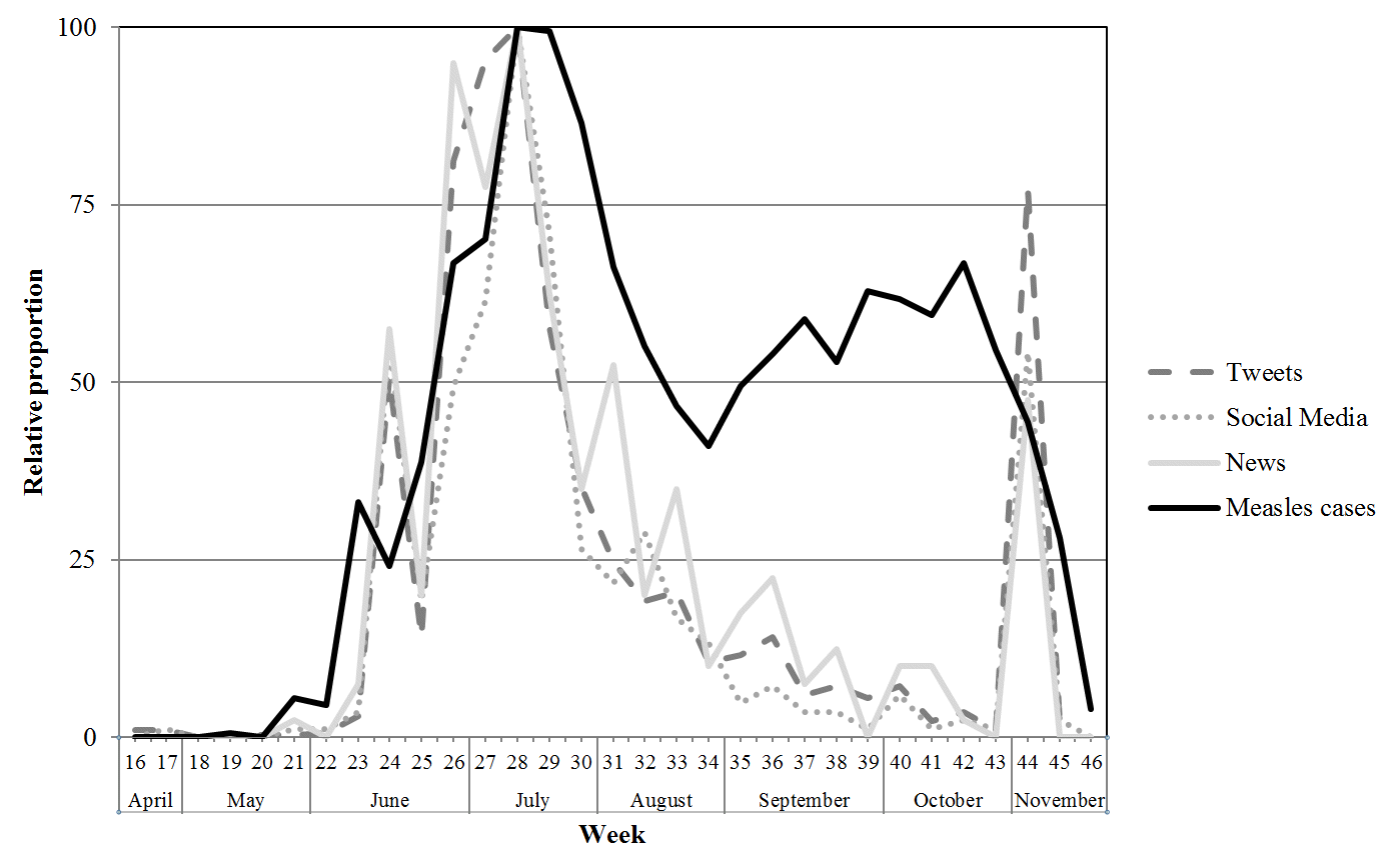
Comparison of relative proportions of weekly tweets, other social media messages, and online newspaper articles to measles cases from April 15 to November 11, 2013. Graph is scaled to the highest peak at week 28 for all 4 data sources (peak assigned a score of 100).

During the measles outbreak, 3 large peaks in the number of messages with a small width were observed for all 3 types of online media data, which coincided with announcements about the measles outbreak by the RIVM and statements made by well-known politicians (Figure 1). The first peak in mid-June coincided with the announcement of the start of the measles outbreak. The second peak in mid-July corresponded with the announcement that additional control measures were to be implemented (ie, additional vaccinations for groups considered most at risk) by RIVM. The second peak also corresponded with public statements made by well-known politicians. The third peak coincided with the announcement that an unvaccinated adolescent had died due to measles complications.

The number of measles cases peaked in mid-July, which was reflected by the peaks in the media reports. However, from the end of August (week 34: 73/2378, 30.70%, 95% CI 24.31%-38.22%) to the end of October (week 42: 119/2378, 50.04%, 95% CI 41.81%-59.37%), a significant increase was shown in the number of measles cases. In the same period, the number of online media messages continued to gradually decrease. Furthermore, after the announcement of the measles-related death on October 28, a steep significant increase from week 43 (eg, for tweets: 3/1982, 0.15%, 95% CI 0.04%-0.41%) to week 44 (for tweets: 234/1982, 11.81%, 95% CI 10.44%-13.28%) in the number of media messages was observed. In the same period, the number of measles cases decreased. Table 3 shows a stronger convergence between the number of social media messages and the number of news messages than between the number of social media messages and the number of reported measles cases.

**Table 3:** Pearson correlations between weekly number of online (social) media messages and weekly number of reported measles cases for the observation period (31 weeks between April 15 and November 11, 2013).

Data source	Tweets		Other social media		Online newspapers	
	*r*	*P*	*r*	*P*	*r*	*P*
Tweets	—	—	—	—	—	—
Other social media	.96	<.001	—	—	—	—
Online newspapers	.96	<.001	.90	<.001	—	—
Reported measles cases	.56	.003	.40	.048	.44	.045

### Manual Topic Analyses

Most tweets and online news articles were about the measles outbreak. Also, most other social media messages addressed the topic measles outbreak but the number did not significantly differ from the number of messages related to other topics. Most retweets addressed the topic of measles prevention, but this was also not statistically significant (Table 4). Overall, perceived risks of measles disease and measles vaccine and refusing vaccination because of religious reasons were also frequently reported topics, but did not significantly differ from the other topics, such as opinions of those who are critical toward vaccination and the trust and role of institutions (eg, government or media).

Comparing the topics of religious- versus nonreligious-oriented newspapers showed that most articles in both types of newspapers were about the measles outbreak and measles prevention (Table 5). The percentages of the topics did not differ significantly between the religious- and nonreligious-oriented newspapers.

**Table 4:** Topics of coded measles-related tweets, retweets, other social media messages, and online newspaper articles.

Topic	Tweets n=136		Retweets n=60		Other social media n=464		Online newspapers n=282		Total N=942	
	n (%)	95% CI	n (%)	95% CI	n (%)	95% CI	n (%)	95% CI	n (%)	95% CI
Measles outbreak	56 (41.2)	33.1-49.6	14 (23)	14-35	95 (20.5)	17.0-24.3	150 (53.2)	47.3-59.0	315 (33.4)	30.5-36.5
Measles prevention	23 (16.9)	11.3-23.9	17 (28)	18-41	92 (19.8)	16.4-23.6	76 (27.0)	22.0-32.4	208 (22.1)	19.5-24.8
Perceived risk	13 (9.6)	5.4-15.4	3 (5)	1-13	90 (19.4)	16.0-23.2	14 (5.0)	2.9-8.0	120 (12.7)	10.7-15.0
Refusing vaccination because of religion	21 (15.4)	10.1-22.3	9 (15)	8-26	58 (12.5)	9.7-15.7	15 (5.3)	3.1-8.4	103 (10.9)	9.1-13.0
Other	16 (11.8)	7.1-18.0	7 (12)	5-22	50 (10.7)	8.2-13.8	3 (1.1)	0.3-2.9	76 (8.1)	6.5-9.9
Critical toward vaccination	3 (2.2)	0.6-5.9	4 (7)	2-15	49 (10.6)	8.0-13.6	16 (5.7)	3.4-8.9	72 (7.6)	6.1-9.5
Trust and role of institutions	4 (2.9)	0.9-6.9	6 (10)	4-20	30 (6.5)	4.5-9.0	8 (2.8)	1.3-5.3	48 (5.1)	3.8-6.6

**Table 5:** Topics of coded measles-related articles in religious- and nonreligious-oriented newspapers.

Topic	Religious newspapers n=79		Nonreligious newspapers n=203		Total N=282	
	n (%)	95% CI	n (%)	95% CI	n (%)	95% CI
Measles outbreak	35 (44)	34-55	115 (56.7)	49.8-63.3	150 (53.2)	47.3-59.0
Measles prevention	24 (30)	21-41	52 (25.6)	20.0-32.0	76 (27.0)	22.0-32.4
Critical toward vaccination	5 (6)	2-13	11 (5.4)	2.9-9.2	16 (5.7)	3.4-8.9
Refusing vaccination because of religious reasons	8 (10)	5-18	7 (3.5)	1.5-6.7	15 (5.3)	3.1-8.4
Perceived risk	5 (6)	2-13	9 (4.4)	2.2-8.0	14 (5.0)	2.9-8.0
Trust and role institutions	0 (0)	0-4	8 (3.9)	1.8-7.3	8 (2.8)	1.3-5.3
Other	2 (3)	0-8	1 (0.5)	0.0-2.4	3 (1.1)	0.3-2.9

### Manual Sentiment Analyses

Sentiment information was most frequently found in tweets (49.19%, 667/1356 messages) and the number of tweets with information differed significantly from the number of tweets expressing other sentiments (see Table 6). In retweets and other social media messages, the sentiment relating to frustration was highest, but the number did not significantly differ from the sentiment information. Overall, the sentiments relating to humor/sarcasm and “other” were expressed in the messages of the different data sources but to a lesser extent than sentiments relating to information and frustration. Sentiments relating to concern, question, minimized risk, relief, and personal experience were hardly expressed in the tweets and other social media messages.

We also analyzed how content was expressed in online news articles and compared religious- with nonreligious-oriented newspapers (Table 7). It was observed that measles-related articles in religious newspapers were more neutral, less alarming, and more reassuring than articles in nonreligious newspapers, but not significantly so. Overall, and within the nonreligious newspapers, the number of neutral and alarming articles was significantly higher than the number of reassuring articles.

We also analyzed the weekly number of messages expressing one of the previously defined sentiments for the 3 data sources, but the numbers were too low to draw conclusions on.

**Table 6:** Sentiments of coded measles-related tweets, retweets, and other social media messages.

Sentiment	Tweets n=1356		Retweets n=626		Other social media n=464		Total N=2446	
	n (%)	95% CI	n (%)	95% CI	n (%)	95% CI	n (%)	95% CI
Information	667 (49.19)	46.53-51.85	214 (34.2)	30.5-38.0	82 (17.7)	14.4-21.3	963 (39.37)	37.45-41.32
Frustration	238 (17.55)	15.60-19.65	232 (37.1)	33.3-40.9	106 (22.8)	19.2-26.8	576 (23.55)	21.90-25.26
Other	123 (9.07)	7.63-10.69	46 (7.4)	5.5-9.6	128 (27.6)	23.7-31.8	297 (12.14)	10.89-13.48
Humor/ Sarcasm	144 (10.62)	9.06-12.34	78 (12.5)	10.0-15.2	46 (9.9)	7.4-12.9	268 (10.96)	9.76-12.24
Concern	59 (4.35)	3.36-5.54	24 (3.8)	2.5-5.6	37 (8.0)	5.8-10.7	120 (4.91)	4.10-5.82
Question	78 (5.75)	4.60-7.09	15 (2.4)	1.4-3.8	13 (2.8)	1.6-4.6	106 (4.33)	3.58-5.20
Minimized risk	23 (1.70)	1.10-2.49	8 (1.3)	0.6-2.4	27 (5.8)	3.9-8.2	58 (2.37)	1.82-3.03
Personal experience	12 (0.88)	0.48-1.50	1 (0.2)	0.0-0.8	19 (4.1)	2.6-6.2	32 (1.31)	0.91-1.82
Relief	12 (0.88)	0.48-1.50	8 (1.3)	0.6-2.4	6 (1.3)	0.5-2.7	26 (1.06)	0.71-1.53

**Table 7:** Sentiments of coded measles-related articles in religious- and nonreligious-oriented newspapers.

Sentiment	Religious newspapers n=79		Nonreligious newspapers n=150		Total N=229	
	n (%)	95% CI	n (%)	95% CI	n (%)	95% CI
Neutral/no sentiment/both alarming and reassuring	37 (47)	36-58	62 (41.3)	33.7-49.3	99 (43.2)	36.9-49.7
Alarming	23 (30)	20-40	69 (46.0)	38.1-54.0	92 (40.2)	34.0-46.6
Reassuring	19 (24)	16-34	19 (12.7)	8.0-18.7	38 (17.0)	12.2-21.8

### Combining the Manual Analyses of Topics and Sentiments

Of the tweets (retweets included) and other social media messages with topics relating to measles incidence or measles prevention, we found that 56.4% (93/165) of messages were informative for measles outbreak and 35.6% (47/132) for measles prevention. In all, 48% (16/33) of the messages with the subtopic of measles-related death within the topic measles outbreak were related to the sentiment of frustration (ie, frustration about persons not vaccinating their child). Of the messages with the topic of refusing vaccination because of religious reasons, we found that 48% (42/88) of the sentiments qualified as frustration.

Of the other social media messages with the topic of perceived risk, we found that 30% (27/90) of messages qualified as minimized risk (ie, in combination with subtopic of measles disease is not severe), 22% (20/90) as concern (ie, in combination with the subtopic of measles disease is severe), and 19% (17/90) as neutral (ie, in combination with the subtopics of adverse events and perceived effectiveness of vaccine). Of the other social media messages with the topic regarding opinions of those who are critical toward vaccination, 43% (21/49) of the messages qualified as neutral and 39% (19/49) as frustration. Of the other social media messages with the topic relating to trust and the role of institutions (eg, government or media), 53% (16/30) of the messages qualified as frustration and 30% (9/30) as neutral.

Both the topic and sentiment were only available for the articles in the religious-oriented newspapers. Of the online newspaper articles with the topic of measles outbreak, we found that 49% (17/35) of the articles qualified as neutral and 46% (16/35) as alarming. Of the articles with the topic regarding measles prevention, 50% (12/24) of the articles qualified as reassuring and 29% (7/24) as neutral. Of the online newspaper articles with the topic of refusing vaccination because of religious reasons, all (n=8) qualified as neutral.

## Discussion

### Principal Findings

The weekly number of social media messages was related more strongly to the number of online news articles than to the number of reported measles cases, supporting the public opinion function of social media more than the disease detection function. In addition, the number of tweets, other social media messages, and online news articles showed a similar distribution over time with 3 large peaks. These findings support the agenda-setting function of the media, showing that the media determine to a large extent what people talk about on social media. Important events with high news values, such as the death of a young girl, resulted in a significant increase in the number of social media messages: people seemed to share their frustration about this measles-related death of a girl who was not vaccinated for religious reasons. The second and largest peak in response to the announcement of additional control measures and statements made by well-known politicians occurred at the same time for both the number of social media messages and reported number of measles cases, but overall patterns between social media and outbreak data diverge.

Particularly interesting is the finding that (social) media attention shows a steep drop after the second peak, whereas the number of reported measles cases remained relatively high. This suggests that the news value of the measles outbreak had dropped and other topics gained prominence. Thus, our findings suggest that social media followed the traditional media agenda for the measles outbreak rather than the measles pattern. It should also be noted that the significant increase in the number of reported measles cases at the end of August may be due to the commencement of schools after the summer break. The spread of the measles virus has been found to occur mostly at schools [1].

Various studies [11-13] showed that estimates of influenza-like illness derived from Twitter accurately track reported disease levels, which is partly the case in our study. Vasterman & Ruigrok [23] showed that it was not the reported number of cases but the number of hospitalizations during the epidemic stage that was in-line with media coverage. Vasterman & Ruigrokl [23] argued that this was probably because almost 50% of the hospitalized patients were children, which made this extra newsworthy. Our findings point to the importance of differentiating between illnesses; for some illnesses, social media may reflect outbreak patterns, whereas for other illnesses social media are more likely to reflect public opinion patterns. Future studies should look further into this issue by examining the role of media hype and news value (eg, it may be that illnesses with low news value such as seasonal flu may more accurately reflect disease levels than illnesses with high news value). It may also be that large international outbreaks follow different rules than smaller local outbreaks. Finally, the present outbreak concerned a specific-risk group, which may also have played a role in the observed patterns.

We also showed that most tweets were about the measles outbreak and were informative, and most newspaper articles were about the measles outbreak and were neutral or alarming. For retweets and other social media, the topics and sentiments were less distinct. Taking all data sources together, the topics of measles outbreak and measles prevention and the sentiments information and frustration were the most present in the messages. People were informing others about the measles outbreak and preventive measures such as vaccination, but also expressed their frustration regarding persons who did not vaccinate because of religious reasons. Some differences were also observed between tweets and retweets. Most tweets were informative, whereas most retweets qualified as frustration. Therefore, it seemed that people found it more important to express their frustration than informing others about the measles outbreak. No significant differences in topic and sentiment were found between religious- and nonreligious-oriented newspapers. Similar to the study by Chew & Eysenbach [14] about H1N1, our study showed that tweets primarily contained news, updates, and information about the outbreak. Chew & Eysenbach [14] also suggested that tweets are a source of experiences. In our study, this was less the case (experiencing measles is a subtopic within the topic measles outbreak) and which was shared more by social media (24/95 messages) than by Twitter (6/70 messages). Regarding sentiments expressed in messages, Chew & Eysenbach [14] found that tweets expressing humor, concern, and questions were the most common, whereas we found the sentiments information and frustration were the most common. The sentiment information is not very surprising because tweets expressed news primarily. Despite that they did not find many informative tweets, they did show that the proportion of tweets containing news increased over time, which was probably because more information about the disease and the vaccine became available. The differences with the study of Chew & Eysenbach [14] might be explained because they analyzed tweets related to an unknown disease and vaccine (ie, H1N1 pandemic) and to a disease spread throughout the entire population. Our study, in contrast, was about a well-known disease and vaccine, and the outbreak mostly affected unvaccinated Orthodox Protestants.

This study did not provide new insights into factors possibly related to intention to vaccination and/or vaccination behavior and could not detect increases or decreases in the number of messages expressing a specific sentiment over time. The fact that we found the leading sentiment was frustration regarding people who refuse vaccinations based on religious grounds might confirm the high vaccination coverage for measles vaccination indicating that our study population favors measles vaccination.

### Limitations

A limitation of this study is that our study population is not well defined, which may underestimate or overestimate the results toward the general public causing a misinterpretation of results. Social media have fast become an important area for the acquisition of new information. Almost 90% of the Dutch population aged 12 years and older use the Internet; of those, 70% are active on social media, particularly Facebook and Twitter (ie, Web 2.0) [25]. It was beyond the scope of this study to identify characteristics of our study population. However, it has been shown that the use of social media does not vary much by gender, ethnicity, and educational level [25], but this may not be the case for those who write about measles on social media. Furthermore, we had no insight into whether the sentiments about the measles outbreak we identified in our data sources were in-line with the sentiment of the general public. More research is needed to ascertain if an association can be found between the topics and sentiments of messages presented in media messages and among the general public. Another remark that has to be made is that we could not get information about the data mining approaches by the companies we retrieved the data from. A last limitation was the relative low kappa for coding sentiments expressed in the social media messages. Overall, social media messages contain larger volumes of text stories and personal experiences compared with tweets and online news articles. This led to difficulties in coding. Sometimes several sentiments were expressed in the same message; in those cases, the first sentiment identified was chosen. Despite these limitations, the biggest advantage of using online data is the continuous data collection and the user-generated content.

### Practical Implications and Future Work

We also wanted to explore whether and how we should monitor the online (social) media data about the NIP for harvesting public opinions possibly related to intention to vaccinate during and in-between outbreaks so that interventions can be made, such as adapting communication to the public. An important real-time worldwide Internet monitor for vaccine concerns that already exists is The Vaccine Confidence Project [26]. However, it could not be used directly for the Netherlands because it does not contain search words in Dutch, but because similar vaccine concerns may be present in various countries, it will be interesting to compare our findings with their findings. Additionally, they developed a typology of concerns and a way to assess the priority of each concern, which might be very useful for us as well as for others. Furthermore, The European Commission’s Joint Research Centre has developed a number of news aggregation and analysis systems (Europe Media Monitor [EMM]) to support EU institutions and Member State organizations with, for example, analyzing real-time news for medical- and health-related topics and providing early warning alerts per category and country. The advantage of this EMM is that it is already an automated process and that it covers many languages, including Dutch. They plan to add an opinion-mining functionality to the existing information extraction components, but this might not be specific enough for our purposes [27].

We believe that real-time monitoring of online (social) media data is important so that the RIVM is aware of the beliefs and opinions of the public about the NIP and is able to detect and respond to possible vaccine concerns in a timely manner. The online (social) media monitoring has an added value to the parental questionnaire sent at regular intervals in the system to monitor the intention and their determinants to vaccinate within the NIP [28] because the online media monitoring generates continuous data and consists of user-generated content. This study addressed various specific topics about a measles outbreak among Orthodox Protestants; therefore, a next step is to explore the public’s opinion about other NIP diseases using similar methodology. Additionally, this study showed that the announcements by the RIVM on their website had a considerable effect on the message volume and posting behavior, which could also be used to generate attention for other health messages related to that particular subject (eg, taking preventive measures). Therefore, the use of these online data may have potential usefulness in public health. In the near future, we will start developing a system that automatically codes messages relating to various NIP diseases. This system would enable the analysis of large amounts of data and allow detection of differences in thoughts and emotions people share on the Internet and will provide insight into user’s characteristics.

### Conclusions

The number of social media messages was related more strongly to the number of online news articles than to the number of reported measles cases. Furthermore, the number of tweets, other social media messages, and online newspaper articles showed a similar distribution over time with 3 large peaks. The peaks in the number of online news articles could very well be explained by announcements by the RIVM (ie, start of the outbreak, additional vaccinations, and a measles-related death) and statements made by well-known politicians. Most messages were about informing people about the measles outbreak and the leading sentiment was frustration regarding people who do not vaccinate for religious reasons. Monitoring online (social) media might be useful for RIVM in deciding whether and how to respond to the public about infectious disease outbreaks. Additionally, the data provide insight into the opinions of the public about infectious diseases outbreaks, which could enable the RIVM to respond appropriately to possible concerns.

## Abbreviations

EMM: Europe Media Monitor
NIP: National Immunization Program
RIVM: The National Institute for Public Health and the Environment
